# A Cascade Graph Convolutional Network for Predicting Protein–Ligand Binding Affinity

**DOI:** 10.3390/ijms22084023

**Published:** 2021-04-14

**Authors:** Huimin Shen, Youzhi Zhang, Chunhou Zheng, Bing Wang, Peng Chen

**Affiliations:** 1 National Engineering Research Center for Agro-Ecological Big Data Analysis & Application, School of Internet & Institutes of Physical Science and Information Technology, Anhui University, Hefei 230601, China; ahu0086@163.com; 2 School of Computer and Information, Anqing Normal University, Anqing 246133, China; 3 School of Computer Science and Technology, Anhui University, Hefei 230601, China; zhengch99@126.com; 4 School of Electrical and Information Engineering, Anhui University of Technology, Ma’anshan 243032, China; bwang@ahut.edu.cn

**Keywords:** graph convolutional network, protein–ligand binding affinity, PDBbind

## Abstract

Accurate prediction of binding affinity between protein and ligand is a very important step in the field of drug discovery. Although there are many methods based on different assumptions and rules do exist, prediction performance of protein–ligand binding affinity is not satisfactory so far. This paper proposes a new cascade graph-based convolutional neural network architecture by dealing with non-Euclidean irregular data. We represent the molecule as a graph, and use a simple linear transformation to deal with the sparsity problem of the one-hot encoding of original data. The first stage adopts ARMA graph convolutional neural network to learn the characteristics of atomic space in the protein–ligand complex. In the second stage, one variant of the MPNN graph convolutional neural network is introduced with chemical bond information and interactive atomic features. Finally, the architecture passes through the global add pool and the fully connected layer, and outputs a constant value as the predicted binding affinity. Experiments on the PDBbind v2016 data set showed that our method is better than most of the current methods. Our method is also comparable to the state-of-the-art method on the data set, and is more intuitive and simple.

## 1. Introduction

The mutual recognition and binding of proteins and ligands occurs in almost all basic biological activities and plays a very important role in these activities. One of the challenges in the field of drug discovery is to find small ligand molecules with high affinity to target proteins in a broad ligand space [1]. Among methods for computing binding affinities between proteins and ligands, biological/chemical experiments and direct calculation based on the first principle of physics or quantum mechanics undoubtedly perform the best [2,3,4]. However, these methods are so time-consuming and costly that they are not suitable for large-scale molecular screening in the early stages of drug discovery. In order to solve this problem, the virtual screening method emerged [5]. In large-scale virtual screening, the docking postures of protein and ligand are selected by a molecular docking program and then scored by the scoring function. The score reflects the affinity between the protein–ligand complex. The scoring function in the docking program is the research focus of affinity evaluation [6,7]. With the development of the early classical scoring function and the use of machine learning and deep learning algorithms to build a model for prediction, the accuracy is constantly improving but has still failed to achieve satisfactory results [8,9].

The research into the scoring function began in the 1990s. Since then, many calculation methods based on different assumptions and rules have been developed. According to the literature [10], the existing calculation methods can be roughly classified into four categories. First, the physics-based scoring function, which is based on force fields, sums up different interaction terms among molecules, including the van der Waals force, electrostatic interaction, hydrogen bond, etc. Earlier versions of docking programs DOCK [11] and AutoDock [12] used this type of scoring functions based on the AMBER force field [13]. Second, empirical scoring functions, which rely on the combined free energy, can be obtained from the weighted sum of different energy terms, and the weight coefficients of each energy term can be obtained according to the fitting relationship between experimental data and the structure of the complex (generally multiple linear regressions). Examples of such methods are ChemScore [14], X-Score [15], and Glide-Score [16]. Third, knowledge-based potential scoring functions are theoretically based on the anti-Boltzmann statistical mechanics method, which is the inverse form of Boltzmann law. Only the structural data are statistically analyzed to calculate binding affinity, where the frequency of atom–atom contact is an important part to measure the binding affinity. The frequency of different atom pairs in the crystal structure in a specific range can be transformed into the potential of atom pair interaction. Examples of such methods are DrugScore [17] and IT-Score [18]. The above three kinds of methods are called classical scoring functions in the current mainstream literature. The fourth one, descriptor-based scoring functions, represents the development trend of scoring functions. Here, protein–ligand interaction or complex structure is encoded by a series of descriptors, and then uses a machine learning or deep learning algorithm with high learning ability to identify the potential patterns hidden in the descriptors, so as to establish a computational model. Typical examples of such methods are RF-Score [19,20] and NNScore [21,22]. RF-Score is characterized by the number of occurrences of specific protein–ligand atom types in a specific distance range, and trained by a random forest algorithm (multiple decision tree integration). NNScore also uses atomic pairs as features, but uses an artificial neural network (the early simple full connection layer network) as the training algorithm. In recent years, with the growth of protein–ligand binding data and the continuous development of deep learning technology, many advanced methods for predicting protein–ligand binding affinity have been developed. Convolutional neural networks have been proved to be effective in image recognition, speech processing, and language translation. Researchers have applied this powerful learning tool in many bioinformatics studies [23,24], including binding affinity prediction of protein-ligand.

José Jiménez et al. [25] voxelize the three-dimensional structures of protein and ligand molecules. Each voxel was represented by descriptors, similar to the 3D image processing in computer vision, and then input into the 3D convolution neural network for training. Pafnucy [26] cuts structures of complexes into 20 Å cubes around the geometric center of the ligand molecule, voxelized it with every 1 Å resolution, described the chemical information of each atom with a 19-dimensional vector and input it into the CNN network. OnionNet [27] divides protein structures with a radius of 30.5 Å around each ligand atom into onion-like hierarchies, calculates the number of specific atom pairs in each layer (contact number), and finally gets 3840 features, which are input into the CNN for training.

In addition to regularizing the molecular structure to use the CNN, there is another solution to make irregular molecule suitable for neural networks, which is to treat molecules as graph data. AGL-Score [28] proposes multiscale-weighted labeled algebraic subgraphs to represent molecules, and then uses the eigenvalues and eigenvectors of the Laplacian matrix and adjacency matrix calculated by subgraphs to describe molecules, and then inputs them into gradient-boosting trees to train the final model. The advantage of this method is that it requires simple information, and it does not need a molecular analog electric field, which may introduce noise. However, the disadvantage of this method is that the information contained in the subgraph is limited and each matrix needs to calculate the eigenvalue and eigenvector. Except for the combination of the graph and machine learning algorithm in recent years, new end-to-end convolutional neural network methods have also been developed [29,30,31,32,33]. Graph convolutional neural network has shown good performance on many biological and chemical calculation problems [34], also including a preliminary attempt in the prediction of protein–ligand binding affinity. Joseph Gomes et al. [35] propose ACNN (a CNN method), which uses the graph convolutional method as a baseline comparison. The specific graph convolution method they used was the early graph convolutional method proposed by Duvenaud and Maclaurin [36]. PotentialNet [37] is a graph neural network-based method for predicting molecular properties and performs well in predicting binding affinity between protein and ligand. Besides chemical bonds, the method also takes Euclidean distance between atoms into account for defining edges in graphs.

According to the above description, we can see that which category of scoring functions represents the right direction has not been agreed on by the academic community. Therefore, advanced computation methods and perspectives are needed in the study of the binding affinity between protein and ligand. As a new technology, graph convolutional neural network performed well in many fields. This work proposes a new cascade convolution calculation method, which learns the graph-embedded representation of proteins and ligand molecules in stages to build a model for protein–ligand binding affinity prediction. We represent the molecule as a graph, and then use a simple linear transformation to deal with the sparsity problem of the one-hot encoding of the original data. The first stage uses the ARMA graph convolutional neural network to learn the characteristics of the atomic space in the protein–ligand complex. In the second stage, one of the variants of the MPNN graph convolutional neural network framework introduces chemical bond information and interacts with atomic features. Finally, it passes through the global add pool and the fully connected layer, and outputs a constant value as the predicted binding affinity. Experimental results showed that the proposed method is comparable to state-of-the-art method used in the PDBBind dataset2016.

## 2. Results and Discussion

### 2.1. APMNet Results

We evaluated our proposed method on PDBBind v2016 and CASF-2013. The model was trained on the training data set, and the hyperparameters of the model were fine-tuned by grid search according to the results on the validation data set, and the model was tested on the core set of PDBbind v2016 and dataset CASF2013. During the training process, we used Pytorch and PytorchGeometric framework for network training, while OpenBabel and rdkit were used for molecule graph processing. The learning rate was 10^−4^ at the first step, and then changed in the step size of 20. The method was implemented using the Adam optimization algorithm and Glorot parameter initialization. The training procedure was performed using Nvidia GeForce 1080 Ti GPU. SmoothL1Loss was used as the loss function for training the model because it has the advantages of L2 Loss and L1 Loss at the same time, which is suitable for regression problems.

**Figure 1:**
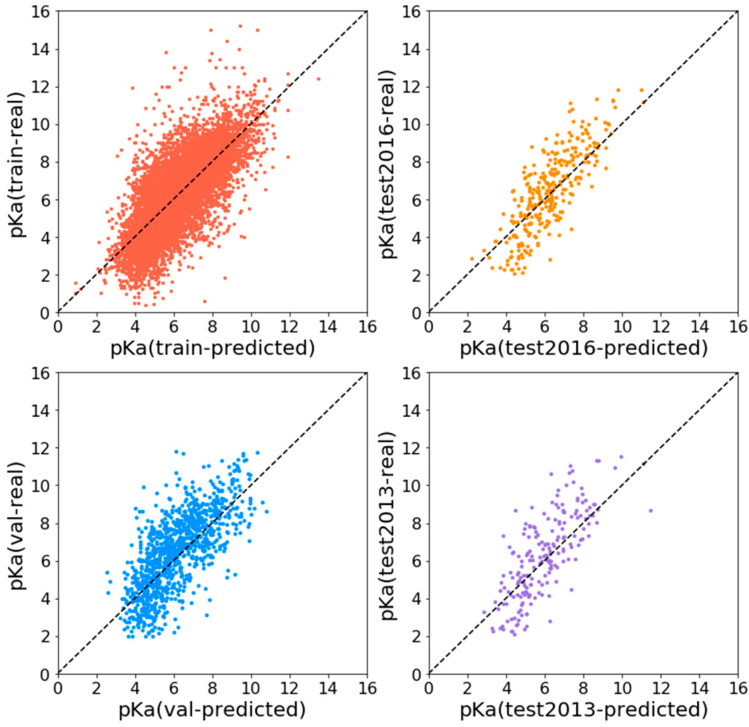
Scatter plots of predicted and true values on different data sets.

Experiments show that APMNet achieves good results on the PDBBind data set, as shown in Table 1. For the 2016 test set, Pearson R is 0.815, RMSE is 1.268, and MAE is 0.998; while for the CASF2013 test set, Pearson R is 0.765, RMSE is 1.508, and MAE is 1.227. The predicted pKa value is highly correlated with the real one. The distribution of predicted and true pKa values of train set and different evaluation set is shown in Figure 1.

### 2.2. Model Analysis

In this section, experiments to analyze the performance of the model were completed. The purpose of these experiments is to explore the influence of different parameters on model performance. The first one is the transformation dimension after molecular input. As mentioned above, because the basic molecular information is input into APMNet model in the form of graph data, the 75-dimensional vector created by one-hot coding is relatively sparse, which is not conducive to subsequent calculation. Our solution is to make a simple linear transformation for them and change their dimensions at the same time. In linear transformation, the amount of dimension change will affect the performance of the model, which is the amount of weight coefficient of the basic information for the molecule. In order to investigate the influence of the dimension change on model performance, we set the dimension respectively to (64, 128, 256), kept the other parameters fixed, and observed the change in training the model.

The second part is the number of ARMA layers and of MPNN layers in the network. According to the experience of neural networks in previous literature, in general, deeper networks can extract more elaborate features, thereby obtaining better prediction performance. However, the method of graph convolution is quite different from those of the conventional convolution method. Experiences in previous work may not be applicable to the graph convolutional neural network. In order to test the influence of convolution layers on the performance of the model, we set the ARMA layers as (3, 5, 7), MPNN layers as (1, 3, 5), and observed the training of the model without changing other parameters.

**Figure 2:**
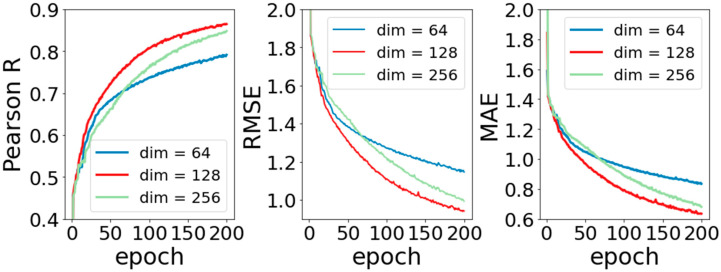
Curves of Pearson R, Root Mean Square Error (RMSE), and Mean Absolute Error (MAE) for different dimension.

Figure 2 shows the experimental results by setting the changed dimensions to 64, 128, and 256. It can be seen that when the dimension is 128, the model performs similarly to that with dimension 256, and both of them perform better than when the dimension is 64. This may be because when the dimension is 64, part of the molecular information is also eliminated for the data being dimensionally reduced. The missing information makes the prediction performance of the model worse, that is to say, when the dimension is higher, the amount of information that can be saved is larger. However, it should be noted that it does not always lead to better performance for the higher dimension. The model with dimension 256 does not perform as well as that with dimension 128. This is because data at too high dimensions creates too many weight coefficients, and the redundant weight coefficients become the noise of the model, which makes the prediction performance worsen.

**Figure 3:**
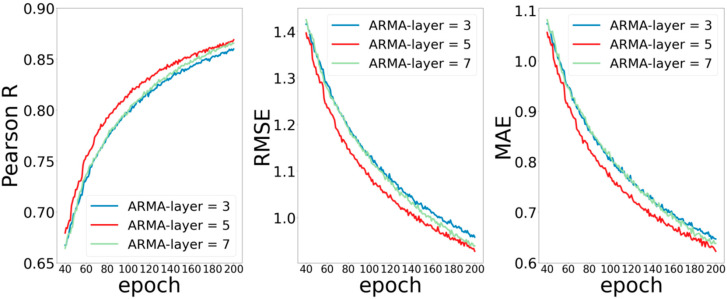
Curves of Pearson R, RMSE, and MAE for different ARMA layers.

Figure 3 shows the experimental results by setting the number of ARMA network layers to 3, 5, and 7. It can be seen that the ARMA model with 5 layers performs better than that with 3 or 7 layers, and the performance of ARMA model with 3 layers is very close to that of ARMA model with 7 layers. Poor performance occurs when the number of layers is 3, which is easy to understand because the network is too shallow to capture enough data features. Similarly, poor performance for the model with 7 layers may be because the increased depth network increases the training error.

Moreover, each layer of ARMA is localized in node space, and its computational complexity is linear in the number of edges. A large number of edges in protein macromolecules makes the number of traceable parameters in the ARMA network layer increase dramatically, and the difficulty of training increase, while the deepening of network will lead to the accumulation of training errors and affect the performance of the model.

**Figure 4:**
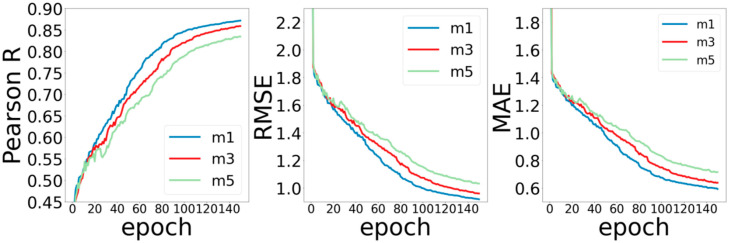
Curves of Pearson R, RMSE, and MAE for different MPNN layers.

Figure 4 shows the experimental results by setting the number of MPNN network layers to 1, 3, and 5. For the Pearson R metric, the model with 1 MPNN layer performs the best, and the model with 5 MPNN layers performs the worst. It may be related to the nature of MPNN calculation. The calculation process of each layer of MPNN itself includes three stages of information (message passing, updates, and aggregation), which is already a complete process for feature extraction. Therefore, increasing the number of layers does not lead to gains in improving the performance of the model. For RMSE and MAE, the increase for the number of layers does not improve the performance of the model.

### 2.3. Ablation Study

We also investigated individual effects for the two main constituents of APMNet architecture. First, we conducted an ablation test for the effect of the ARMA layer by dropping the MPNN layer. Second, we assessed the effect of the presence of the MPNN layer by dropping the ARMA layer. Finally, we ran the whole neural network for comparison with the two individual constituents.

**Figure 5:**
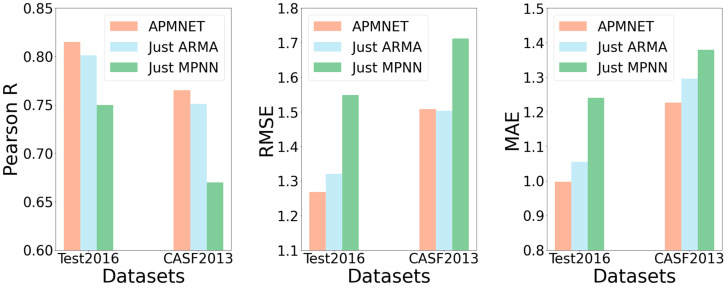
Performance with Pearson R, RMSE, and MAE for different ablation test model.

Figure 5 shows the performance comparison of the ablation tests on the two test datasets, Test2016 and CASF2013, with different models. In Figure 5, we show that APMNet outperformed the other two methods on the two test datasets, except the performance of CASF2013 in measuring RMSE, which is only slightly worse than ARMA. Accordingly, it can confirm the necessity of the combination of the two constituents.

### 2.4. Performance Comparison with Other Scoring Function Methods

In order to verify the effectiveness of the proposed method, the proposed method was compared with other methods (including classic methods and deep learning methods) on the two test sets, Test2016 and CASF2013. Table 2 records the results of Pearson R and RMSE for each method on the Test2016 dataset. The methods listed in the table are those ones that currently perform well in protein–ligand affinity prediction. On the test2016, KDeep [25] and Pafnucy [26] both cut the three-dimensional structure of protein ligands into a regular cubic structure, and then combined them with a grid chemoinformatics descriptor, using 3D-CNN to train the model. This type of method represents the traditional idea of regularizing the molecular structure. Although they perform well, the required characteristics are complex and rely on chemical assumptions. For example, the simulated molecular force field does not necessarily conform to the facts and may introduce a lot of noise. RF-Score [19] and OnionNet [27] counted the number of atomic contacts within a certain distance as the feature. The former model is based on random forest and the latter one is based on CNN. There is a problem among these methods in that the conformational difference of the same molecule will affect the prediction results. This problem no longer exists in graph-based methods, because the three-dimensional conformation of molecules is not needed when treating molecules as graphs.

As the AGL-Score [28] results show, the graph-based method is superior to other methods in Pearson R and RMSE. However, AGL-Score only uses graphs to represent molecules. Each subgraph needs to calculate eigenvectors and eigenvalues as features, and then input the information into machine learning models to make feature selections. Our method goes further than the AGL-Score, which learns features directly on the graph and makes the process more straightforward. In addition, the two graph convolution methods are complementary to each other, so as to learn the underlying rules hidden in the structure of protein–ligand complexes more efficiently. It can be seen that in terms of RMSE, APMNet, KDeep, AGL-Score, and OnionNet methods perform similarly, and they all show good prediction performance. The RMSE value of APMNet is the lowest, indicating that the prediction error of our method is the lowest. In terms of Pearson R, APMNet has also achieved competitive performance with other methods. Although slightly lower than AGL-Score, the APMNet method is simpler and more intuitive.

Table 3 records the results of Pearson R and SD for each method on the CASF2013 dataset. As can be seen from the table, the performance of classic methods (X-Score, ChemScore, ChemPLP, AutoDock Vina, and AutoDock) is worse than machine learning or deep learning methods (OnionNet, RF-Score-v3, Pafnucy, AGL-Score, kNN-Score, and APMNet). This may be because classic methods use fewer data features and are only applicable to small data sets. The deep learning method performs better than the machine learning method perhaps because the end-to-end deep learning method is less affected by the limitation of hand-crafted features, which do not need feature design and selection.

In addition, the CASF2013 test set also provides the ranking power capability of test benchmarks. Ranking power refers to the ability to correctly rank the known ligands of a specific target according to binding affinity. On the CASF2013 data set, the measure for evaluating the ranking power is the accuracy of correctly sorting the three complexes in each cluster in the entire data set according to their binding affinity values. Table 4 records the comparison results of the ranking power of the APMNet method and other methods. The high level in the table means that the sorting of the three data is completely correct, and the low level means that as long as the maximum value can be correctly predicted, it can be counted as correct, regardless of whether the median value and the minimum value are sorted correctly. It can be seen that the ranking power of the APMNet method performs the best regardless of the high level or the low level, which further illustrates the superiority of our method.

## 3. Dataset and Method

### 3.1. Dataset Preparation

The data used in this paper is from the PDBBind database [41], where the version of V2016 was selected for comparison with other methods on the premise that the amount of data is large enough. Protein–protein complexes, protein–nucleic acid complexes, and nucleic acid–ligand complexes were removed; only protein–ligand complex data was retained. The original data uses “pdb id” to represent the protein–ligand complex, in which the protein and ligand structure data are recorded with their respective chemical connection tables (in pdb and mol2 or sdf format). The data set is divided into three levels, general set, refined set, and core set. The general set includes all the data. The refined set contains the high-quality data selected from the general set according to some rules, whose structural resolution is higher than the general set. The core set is selected from the refined set. It is obtained by systematically sampling from the similarity clustering on the proteins in the refined set.

To implement the binding affinity prediction, the data was divided into training, validation, and test sets. Consistent with other methods, the test set in this method was selected from a total of 290 complexes in the core set, and then the validation set was randomly selected from 1000 complexes in the refined set. All the remaining complexes were used as the training set. In addition, the CASF-2013 Benchmark dataset [42] was used as an additional test set. This dataset was also proposed by the PDBBind authors, and was used for performance comparison between different methods. CASF-2013 has 87 duplicate data with the training set, so these 87 complexes were removed from the training set to ensure that there was no data intersection between the test set and the training and validation sets. The binding affinity (*k_i_*, *k_d_*, *IC*_50_) used for labeling samples in the model were all from the original data set. We chose the form of *pka*(−*log*_10_*K_x_*) as the prediction target.

Before inputting molecular information into the network, we first used Open Babel to convert the mol2 format of the ligand molecule into pdb format, and then used RDKit to read the molecular information. Moreover, some molecules could not be processed by RDKit because of atoms with unreasonable explicit valence, which were discarded in the training set. The final data set consisted of 11,844 samples for the training set, 1000 for the validation set, 290 for test2016, and 195 for CASF-2013. Specific data id can be found in Supplementary File S1.

### 3.2. Graph Embedding of Protein–Ligand Complexes

A graph is a kind of data structure that is suitable for characterizing molecules. Nodes and edges are two constituent structures respectively corresponding to the 2D atomic and chemical bonds of molecules. Unlike the voxelized method, which limits a regular cube range, the number of nodes and edges in the graph are unlimited, and molecules with different sizes can be represented flexibly. The graph convolutional neural network, as a form designed to deal with structures of irregular topological relationship, needs molecular data to be represented as graphs before being input to the network. A molecular graph is composed of node feature vectors, edge feature vectors, and connection relationships between nodes. The connection relationships between nodes are represented by an adjacency matrix *A*, *A* ∈ *R^n×n^*, where *n* represents the number of nodes. If there is a chemical bond between atom *i* and atom *j*, then *A_ij_* = 1, otherwise *A_ij_* = 0. The adjacency matrix *A* is used to calculate a Laplacian matrix *L*, where *L* is used to calculate the ARMA convolution.

Suppose that node information matrix *X* represents all the atomic information of one molecule, *X* ∈ *R^n×m^*, where *n* represents the number of nodes (atoms), and *m* represents the dimension of the feature vector of the nodes. The feature vectors of atoms and chemical bonds in this article are the same as the graph features in the DeepChem [43] package. The feature vector of each atom consists of element type, chemical valence, form charge, free radical electron, hybrid orbit, and aromatic one-hot code. Table 5 lists the total of 75 features for detailed description. The description labeled one-hot is composed of 0 and 1, and the descriptions that are not labeled one-hot, such as Formal Charge and Radical Electrons, are represented by their specific values. The edge (chemical bond) feature vector in the graph contains 6 types of chemical bonds, namely single bonds, double bonds, triple bonds, aromaticity, conjugation, and whether they are rings. We treated protein molecules and ligand molecules equally, and denoted them as *Xpocket* and *Xligand* respectively according to the above rules. In order to study the binding relationship between protein and ligand, the node feature matrix *Xpocket* of proteins and matrix *Xligand* of ligand molecules were concatenated for representing protein–ligand complex’s nodes. Afterwards, we had *X* represent the protein–ligand complex.

### 3.3. Neural Network Architecture

To learn potential features from protein–ligand complexes, the protein–ligand complex is represented as a graph and then input to a graph convolutional neural network. The network structure of this paper consists of four parts, two linear transformations, and two types of graph convolution calculations.

**Figure 6:**
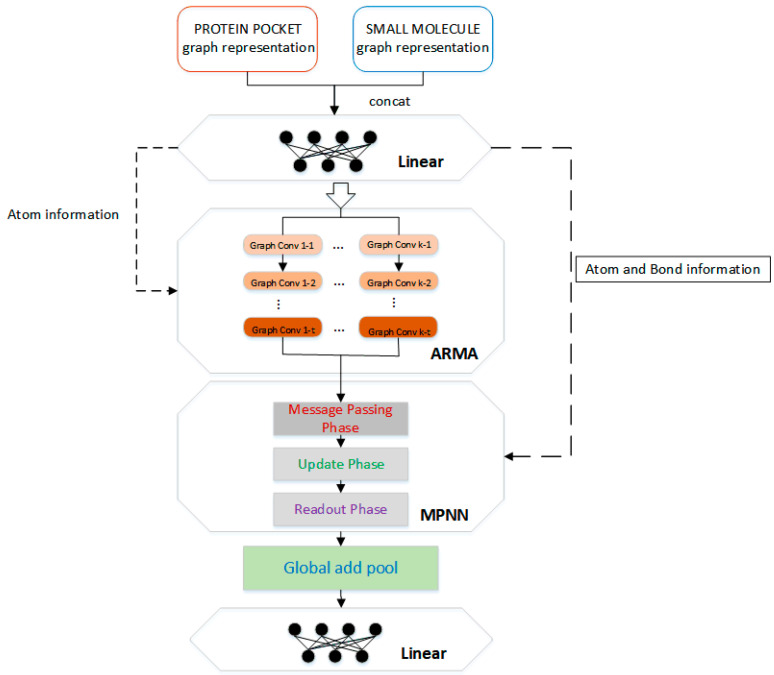
Schematic representation of the APMNet workflow.

The architecture of APMNet (ARMA Plus MPNN neural Network) is summarized in Figure 6. First, a simple linear transformation is performed on the atomic information matrix *X* of the protein–ligand complex. The reason of the transformation is that graph *X* is encoded by one-hot atomic basic information, which is sparse and not conducive to model training. We have the transformed feature vector as *X’* ∈ *R^n×m1^*, where *n* represents the number of atoms in the complex (protein and ligand) molecule, and *m1* represents the transformed feature dimension.

Next is the graph convolutional neural network, which integrates the ARMA [44] graph convolution calculation method and the graph convolution calculation method described in the MPNN framework [45]. The ARMA layer is a graph convolutional layer based on an auto-regressive moving average filter. Compared to mainstream polynomial filters [46,47,48] that are defined in the spectral domain, it breaks through some inherent limitations to make it more robust and has shown good results in a variety of downstream tasks. In addition, the recursive implementation of the ARMA neural network solves the scalability problem of the traditional ARMA filter, making it localized in node space, and has stronger modeling capabilities in node space. The calculation is as follows:

Equation (1):

(1) $${\bar{X}}_{K}^{\left(t+1\right)}=\sigma (\tilde{L}{\bar{X}}^{t}W^{t}+XV^{t}),\;\tilde{L}=I-L,\;X^{\prime }=\frac{1}{k}\sum_{k=1}^{K}{\bar{X}}_{K}^{T}$$

where *L* is computed from adjacent matrix *A* and degree matrix *D*, *L = D^−1/2^AD^−1/2^*, *I* is the identity matrix, *W* and *V* are trainable parameters, *X* is the initial node features, *K* is the number of parallel stacks, *T* is number of layers, and σ is the activation function.

The main part of the molecular information graph is atom information, and the information between atoms interacts in the ARMA layer, which is conducive to the learning of structural features related to the binding affinity between protein and ligand. In addition, although the chemical bond characteristic (edge) accounts for a relatively small amount of information, it is also an important part of intermolecular binding. Note that although there are some non-covalent interactions between proteins and ligands, they are ignored in the model input and only these bonds in the proteins and the ligands are calculated separately. The reason is that the more common calculation scenario in practical applications is to predict the binding affinities between proteins and ligand molecules which are not bound together. Followed by the ARMA convolution layer, we added another graph convolution layer that can learn edge features, which comes from one implementation of the MPNN framework. In MPNN, the edge feature is input into the ordinary neural network. After linear changes and non-linear activation functions, it interacts with the node feature to learn features through information transfer, update, and aggregation between neighboring nodes.

According to Gilmer et al. [45], the MPNN framework consists of three phases: (1) the message passing phase, (2) the update phase, and (3) the readout phase. The message function (Mt) is carried out on the graph elements (hidden state of atom *i*, atom *j*, and edge between atom *i* and *j*) to create message information *m_i_^t+1^* in the message passing phase (Equation (2)). The update phase is performed for each node *i* as a result of applying an update function to the hidden state vector *h* and the newly computed message information vector *m_i_^t+1^* (Equation (3)). The readout phase computes a feature vector for the whole graph by some readout function *R* (Equation (4)). Equations (5) and (6) represent one specific implementation of the MPNN framework in our work. The specific calculation equations are as follows:

(2) $$m_{i}^{t+1}=\sum_{j\in N(i)}M_{t}(h_{i}^{t},h_{j}^{t},e_{ij}^{})$$

(3) $$h_{i}^{t+1}=U_{t}(h_{i}^{t},\;m_{i}^{t+1})$$

(4) $$\overset{⌢}{y}=R(\{h_{i}^{T}|i\in G\})$$

(5) $$x_{i}^{\prime }=x_{i}+\sum_{j\in N(i)}x_{j}•(e_{ij})$$

(6) $$X_{graph}^{}=\sum_{i}^{n}x_{i}^{\prime }$$

where *x_i_* and *x_j_* correspond to atom *i* and *j*, respectively, *N(i)* denotes neighbors of atom *i*, *e_ij_* corresponds to bond edge vector between atom *i* and *j* and denotes a neural network, *n* is number of atoms.

The atomic information and chemical bond information in the protein–ligand complex molecule are calculated by two types of convolution networks, then, the calculation result is passed through the global add pool for downsampling and the fully connected layer for vector dimension change, and finally a constant value is output as the predicted binding affinity.

### 3.4. Evaluation Metrics

We adopted three commonly used metrics in regression tasks to evaluate the performance of the model. They are the Root Mean Square Error (RMSE), Pearson correlation coefficient, and Mean Absolute Error. These metrics are calculated as follows (7)–(9):

(7) RMSE=1n∑i=1n(yi′−yi)2

(8) $$\mathrm{Pearson}\;R=\frac{\sum \left(y_{i}-{\bar{y}}_{i})(y_{i}^{\prime }-{\bar{y}}_{i}^{\prime }\right)}{\sqrt{\sum {\left(y_{i}-{\bar{y}}_{i}\right)}^{2}\sum {\left(y_{i}^{\prime }-{\bar{y}}_{i}^{\prime }\right)}^{2}}}$$

(9) $$\mathrm{MAE}=\frac{1}{n}\sum_{i=1}^{n}|y_{i}^{\prime }-y_{i}|$$

where $y_{i}^{\prime }$ is the predicted binding affinity, $y_{i}$ is the real binding affinity, ${\bar{y}}_{i}$ and ${\bar{y}}_{i}^{\prime }$ refer to the averages of the predicted value and the experimental value, respectively.

The RMSE is the standard deviation of the prediction errors, which is a measure of how spread out these prediction errors are. The Pearson correlation coefficient measures the linear relationship between predicted values and real values, which value varies between −1 and +1, with 0 implying no correlation. Correlations of −1 or +1 imply an exact linear relationship. Positive correlations imply a positive correlation between the predicted values and the real values; the larger value of Pearson R illustrates that the model is better. Mean Absolute Error (MAE) is used to measure the error between the predicted values and the real values.

## 4. Conclusions

Calculation of the binding affinity between protein and ligand is an extremely important part of drug discovery. In order to accurately predict the binding affinity between protein and ligand, this paper proposes a new graph-based neural network, using more intuitive and simple feature descriptors (the basic information of atoms in the molecular structure) to learn affinities from molecular structure in stages. The advantage of our method is in that the required features are simple, needing no molecular force field or three-dimensional molecular conformation and no complicated preprocessing or optimization of molecular structure files. Experimental results show that our model achieves a good performance on the same test set in comparison with the other deep learning-based scoring functions and the classic scoring functions.

## Figures and Tables

**Table 1:** APMNet’s performance.

Dataset	Pearson R	RMSE	MAE
Test2016	0.815	1.268	0.998
CASF2013	0.765	1.508	1.227

**Table 2:** Performance Comparison of different methods on Test2016.

Test2016	Scoring Functions	Pearson R	RMSE
	APMNet	0.815	1.268
	OnionNet [27]	0.816	1.278
	KDeep [25]	0.82	1.27
	RF-Score-v3 [19]	0.80	1.39
	Pafnucy [26]	0.78	1.42
	AGL-Score [28]	0.833	1.271

**Table 3:** Performance comparison of different methods on CASF2013.

CASF-2013	Scoring Functions	Pearson R	SD
	APMNet	0.77	1.44
	OnionNet [27]	0.78	1.45
	RF-Score-v3 [19]	0.74	1.51
	Pafnucy [26]	0.70	1.61
	AGL-Score [28]	0.792	1.271
	kNN-Score [38]	0.672	1.65
	X-Score [38]	0.614	1.78
	ChemScore [38]	0.592	1.82
	ChemPLP [38]	0.579	1.84
	AutoDock Vina [39]	0.54	1.90
	AutoDock [39]	0.54	1.91

**Table 4:** Performance comparison of different methods in the ranking power test.

	Success Rates (%) on Crystal Structures	
	High-Level	Low-Level
APMNet	66.15	78
X-Score^HM^	58.5	72.3
ChemPLP@GOLD	58.5	72.3
PLP2@DS	55.4	72.3
GoldScore@GOLD	55.4	76.9
ChemScore@SYBYL	53.8	67.7
ChemScore@GOLD	46.2	63.1

Note: The data used for comparison in the table comes from the literature [40].

**Table 5:** Atom feature vectors.

Feature Group	Description	Number *
Atom type	C, N, O, S, F, Si, P, Cl, Br, Mg, Na, Ca, Fe, As, Al, I, B, V, K, Tl, Yb, Sb, Sn, Ag, Pd, Co, Se, Ti, Zn, H, Li, Ge, Cu, Au, Ni, Cd, In, Mn, Zr, Cr, Pt, Hg, Pb, unknown (one-hot)	44
Degree	number of directly-bonded neighbors (one-hot)	11
Valence	explicit valence of the atom (one-hot)	7
Formal Charge	electronic charge	1
Radical Electrons	number of radical electrons	1
Hybridization	atom hybridization (one-hot)	5
Aromaticity	is aromatic	1
Hydrogens	the total number of Hs (explicit and implicit) on the atom (one-hot)	5

* The number denotes the number of features in each feature group.

## Data Availability

Not applicable.

## Supplementary Materials

The following are available online at https://www.mdpi.com/article/10.3390/ijms22084023/s1, The final data set consists of 11,844 samples for the training set, 1000 for the validation set, 290 for test2016, and 195 for CASF-2013. Specific data id can be found in Supplementary File S1.

## References

[1] Chan H.S., Shan H., Dahoun T., Vogel H., Yuan S. (2019). Advancing drug discovery via artificial intelligence. Trends Pharmacol. Sci..

[2] Kairys V., Baranauskiene L., Kazlauskiene M., Matulis D., Kazlauskas E. (2019). Binding affinity in drug design: Experimental and computational techniques. Expert Opin. Drug Discov..

[3] Massova I., Kollman P.A. (2000). Combined molecular mechanical and continuum solvent approach (MM-PBSA/GBSA) to predict ligand binding. Perspect. Drug Discov. Des..

[4] Mikulskis P., Genheden S., Ryde U. (2014). A Large-Scale Test of Free-Energy Simulation Estimates of Protein–Ligand Binding Affinities. J. Chem. Inf. Model..

[5] De Azevedo W.F. (2019). Docking Screens for Drug Discovery.

[6] Du X., Yuan-Ling X., Xia Y.-L., Ai S.-M., Liang J., Sang P., Ji X.-L., Liu S.-Q. (2016). Insights into Protein–Ligand Interactions: Mechanisms, Models, and Methods. Int. J. Mol. Sci..

[7] Heck G.S., Pintro V.O., Pereira R.O., Levin N.M.B., de Azevedo W.F. (2017). Supervised machine learning methods applied to predict ligand-binding affinity. Curr. Med. Chem..

[8] Ain Q.U., Aleksandrova A., Roessler F.D., Ballester P.J. (2015). Machine-learning scoring functions to improve structure-based binding affinity prediction and virtual screening. Wiley Interdiscip. Rev. Comput. Mol. Sci..

[9] Li J., Fu A., Zhang L. (2019). An Overview of Scoring Functions Used for Protein–Ligand Interactions in Molecular Docking. Interdiscip. Sci. Comput. Life Sci..

[10] Liu J., Wang R. (2015). Classification of Current Scoring Functions. J. Chem. Inf. Model..

[11] Makino S., Kuntz I.D. (1997). Automated flexible ligand docking method and its application for database search. J. Comput. Chem..

[12] Goodsell D.S., Morris G.M., Olson A.J. (1996). Automated docking of flexible ligands: Applications of AutoDock. J. Mol. Recognit..

[13] Weiner S.J., Kollman P.A., Case D.A., Singh U.C., Ghio C., Alagona G., Profeta S., Weiner P. (1984). A new force field for molecular mechanical simulation of nucleic acids and proteins. J. Am. Chem. Soc..

[14] Eldridge M.D., Murray C.W., Auton T.R., Paolini G.V., Mee R.P. (1997). Empirical scoring functions: I. The development of a fast empirical scoring function to estimate the binding affinity of ligands in receptor complexes. J. Comput. Aided Mol. Des..

[15] Wang R., Lai L., Wang S. (2002). Further development and validation of empirical scoring functions for structure-based binding affinity prediction. J. Comput. Aided Mol. Des..

[16] Friesner R.A., Banks J.L., Murphy R.B., Halgren T.A., Klicic J.J., Mainz D.T., Repasky M.P., Knoll E.H., Shelley M., Perry J.K. (2004). Glide: A New Approach for Rapid, Accurate Docking and Scoring. 1. Method and Assessment of Docking Accuracy. J. Med. Chem..

[17] Gohlke H., Hendlich M., Klebe G. (2000). Knowledge-based scoring function to predict protein-ligand interactions. J. Mol. Biol..

[18] Huang S.Y., Zou X. (2006). An iterative knowledge-based scoring function to predict protein–ligand interactions: I. Derivation of interaction potentials. J. Comput. Chem..

[19] Ballester P.J., Mitchell J.B. (2010). A machine learning approach to predicting protein–ligand binding affinity with applications to molecular docking. Bioinformatics.

[20] Ballester P.J., Schreyer A., Blundell T.L. (2014). Does a more precise chemical description of protein–ligand complexes lead to more ac-curate prediction of binding affinity?. J. Chem. Inf. Modeling.

[21] Durrant J.D., McCammon J.A. (2010). NNScore: A Neural-Network-Based Scoring Function for the Characterization of Protein−Ligand Complexes. J. Chem. Inf. Model..

[22] Durrant J.D., McCammon J.A. (2011). NNScore 2.0: A Neural-Network Receptor–Ligand Scoring Function. J. Chem. Inf. Model..

[23] Le N.Q., Nguyen B.P. (2019). Prediction of FMN Binding Sites in Electron Transport Chains based on 2-D CNN and PSSM Profiles. IEEE/ACM Trans. Comput. Biol. Bioinform..

[24] Le N.Q.K., Yapp E.K.Y., Nagasundaram N., Yeh H.Y. (2019). Classifying promoters by interpreting the hidden information of DNA se-quences via deep learning and combination of continuous FastText N-grams. Front. Bioeng. Biotechnol..

[25] Jiménez J., Skalic M., Martinez-Rosell G., De Fabritiis G. (2018). K DEEP: Protein–ligand absolute binding affinity prediction via 3D-convolutional neural networks. J. Chem. Inf. Modeling.

[26] Stepniewska-Dziubinska M.M., Zielenkiewicz P., Siedlecki P. (2018). Development and evaluation of a deep learning model for protein–ligand binding affinity prediction. Bioinformatics.

[27] Zheng L., Fan J., Mu Y. (2019). OnionNet: A multiple-layer inter-molecular contact based convolutional neural network for protein-ligand binding affinity prediction. arXiv.

[28] Nguyen D.D., Wei G.-W. (2019). AGL-Score: Algebraic Graph Learning Score for Protein–Ligand Binding Scoring, Ranking, Docking, and Screening. J. Chem. Inf. Model..

[29] Bronstein M.M., Bruna J., LeCun Y., Szlam A., Vandergheynst P. (2017). Geometric deep learning: Going beyond euclidean data. IEEE Signal Process. Mag..

[30] Battaglia P.W., Hamrick J.B., Bapst V., Sanchez-Gonzalez A., Zambaldi V., Malinowski M., Tacchetti A., Raposo D., Santoro A., Faulkner R. (2018). Relational inductive biases, deep learning, and graph networks. arXiv.

[31] Zhang Z., Cui P., Zhu W. (2018). Deep Learning on Graphs: A Survey. arXiv.

[32] Zhou J., Cui G., Zhang Z., Yang C., Liu Z., Sun M. (2018). Graph neural networks: A review of methods and applications. arXiv.

[33] Wu Z., Pan S., Chen F., Long G., Zhang C., Yu P.S. (2021). A Comprehensive Survey on Graph Neural Networks. IEEE Trans. Neural Netw. Learn. Syst..

[34] Sun M., Zhao S., Gilvary C., Elemento O., Zhou J., Wang F. (2020). Graph convolutional networks for computational drug development and discovery. Brief. Bioinform..

[35] Gomes J., Ramsundar B., Feinberg E.N., Pande V.S. (2017). Atomic convolutional networks for predicting protein-ligand binding affinity. arXiv.

[36] Duvenaud D.K., Maclaurin D., Iparraguirre J., Bombarell R., Hirzel T., Aspuru-Guzik A., Adams R.P. (2015). Convolutional networks on graphs for learning molecular fingerprints. Advances in Neural Information Processing Systems.

[37] Feinberg E.N., Sur D., Wu Z., Husic B.E., Mai H., Li Y., Sun S., Yang J., Ramsundar B., Pande V.S. (2018). Potentialnet for molecular property prediction. ACS Cent. Sci..

[38] Khamis M.A., Gomaa W. (2015). Comparative assessment of machine-learning scoring functions on PDBbind 2013. Eng. Appl. Artif. Intell..

[39] Gaillard T. (2018). Evaluation of AutoDock and AutoDock Vina on the CASF-2013 Benchmark. J. Chem. Inf. Model..

[40] Li Y., Han L., Liu Z., Wang R. (2014). Comparative Assessment of Scoring Functions on an Updated Benchmark: 2. Evaluation Methods and General Results. J. Chem. Inf. Model..

[41] Liu Z., Su M., Han L., Liu J., Yang Q., Li Y., Wang R. (2017). Forging the basis for developing protein–ligand interaction scoring functions. Acc. Chem. Res..

[42] Li Y., Liu Z., Liu J., Han L., Zhao Z., Wang R. (2014). Comparative Assessment of Scoring Functions on an Updated Benchmark: 1. Compilation of the Test Set. J. Chem. Inf. Model..

[43] Wu Z., Ramsundar B., Feinberg E.N., Gomes J., Geniesse C., Pappu A.S., Leswing K., Pande V. (2018). MoleculeNet: A benchmark for molecular machine learning. Chem. Sci..

[44] Bianchi F.M., Grattarola D., Livi L., Alippi C. (2019). Graph Neural Networks with Convolutional ARMA Filters. arXiv.

[45] Gilmer J., Schoenholz S.S., Riley P.F., Vinyals O., Dahl G.E. Neural message passing for quantum chemistry. Proceedings of the 34th International Conference on Machine Learning.

[46] Henaff M., Bruna J., LeCun Y. (2015). Deep convolutional networks on graph-structured data. arXiv.

[47] Defferrard M., Bresson X., Vandergheynst P. (2016). Convolutional neural networks on graphs with fast localized spectral filtering. Advances in Neural Information Processing Systems.

[48] Kipf T.N., Welling M. (2016). Semi-supervised classification with graph convolutional networks. arXiv.

